# Abrogation of ORF8–IRF3 binding interface with Carbon nanotube derivatives to rescue the host immune system against SARS-CoV-2 by using molecular screening and simulation approaches

**DOI:** 10.1186/s13065-024-01185-4

**Published:** 2024-05-11

**Authors:** Muhammad Suleman, Abduh Murshed, Kashif Imran, Abbas Khan, Zafar Ali, Norah A. Albekairi, Dong-Qing Wei, Hadi M. Yassine, Sergio Crovella

**Affiliations:** 1 Laboratory of Animal Research Center (LARC), Qatar University, Doha, Qatar; 2 Center for Biotechnology and Microbiology, University of Swat, Swat, Pakistan; 3 Department of Intensive Care Unit, Affiliated Hospital of Guangdong Medical University, Zhanjiang, 524000 China; 4 Services Institute of Medical Sciences, Lahore, Pakistan; 5 Department of Bioinformatics and Biological Statistics, School of Life Sciences and Biotechnology, Shanghai Jiao Tong University, Shanghai, 200240 People’s Republic of China; 6 School of Medical and Life Sciences, Sunway University, 47500 Sunway City, Malaysia; 7 Department of Pharmacology and Toxicology, College of Pharmacy, King Saud University, Post Box 2455, 11451 Riyadh, Saudi Arabia; 8 Biomedical Research Center, Qatar University, 2713 Doha, Qatar; 9 College of Health Sciences-QU Health, Qatar University, 2713 Doha, Qatar

**Keywords:** SARS-CoV-2, ORF8, IRF3, Nanotubes, Molecular simulation

## Abstract

The COVID-19 pandemic, caused by the SARS-CoV-2 virus, has led to over six million deaths worldwide. In human immune system, the type 1 interferon (IFN) pathway plays a crucial role in fighting viral infections. However, the ORF8 protein of the virus evade the immune system by interacting with IRF3, hindering its nuclear translocation and consequently downregulate the type I IFN signaling pathway. To block the binding of ORF8–IRF3 and inhibit viral pathogenesis a quick discovery of an inhibitor molecule is needed. Therefore, in the present study, the interface between the ORF8 and IRF3 was targeted on a high-affinity carbon nanotube by using computational tools. After analysis of 62 carbon nanotubes by multiple docking with the induced fit model, the top five compounds with high docking scores of − 7.94 kcal/mol, − 7.92 kcal/mol, − 7.28 kcal/mol, − 7.19 kcal/mol and − 7.09 kcal/mol (top hit1-5) were found to have inhibitory activity against the ORF8–IRF3 complex. Molecular dynamics analysis of the complexes revealed the high compactness of residues, stable binding, and strong hydrogen binding network among the ORF8-nanotubes complexes. Moreover, the total binding free energy for top hit1-5 was calculated to be − 43.21 ± 0.90 kcal/mol, − 41.17 ± 0.99 kcal/mol, − 48.85 ± 0.62 kcal/mol, − 43.49 ± 0.77 kcal/mol, and − 31.18 ± 0.78 kcal/mol respectively. These results strongly suggest that the identified top five nanotubes (hit1-5) possess significant potential for advancing and exploring innovative drug therapies. This underscores their suitability for subsequent in vivo and in vitro experiments, marking them as promising candidates worthy of further investigation.

## Introduction

The current COVID-19 pandemic, caused by SARS-CoV-2, is genetically connected to the SARS-CoV virus responsible for severe acute respiratory syndrome in 2002–2003, as well as other SARS-related viruses found in bats [1]. SARS-CoV-2’s proteome consists of 12 distinct ORFs (open reading frames), which give rise to four structural proteins and twenty-two non-structural proteins [2]. Among the structural proteins are the nucleocapsid (N), membrane (M), envelope (E), and spike (S), while the non-structural proteins are encoded by ORF1ab and six accessory proteins (ORF3a, ORF6, ORF7a, ORF7b, ORF8, and ORF9), encompassing nsp 1–16 [3].

In the human defense system, the activation of the type 1 interferon (IFN) pathway contribute to abolish viral infection. The host pattern recognition receptors are actively involved in the identifications of pathogen-associated molecular patterns and activation of Interferon regulatory factor 3 (IRF3) [4]. In general, the IRF3 is normally present in the cytoplasm in an inactive state, however, during viral pathogenesis, the IRF3 is activated by phosphorylation and translocated to the nucleus [5]. In the nucleus, the activated IRF3 binds to conserved sequences known as IFN stimulated response elements to trigger the transcription of type I IFN genes which are necessary for the control of early viral infection [6, 7].

For effective infection, viruses, particularly coronaviruses, have developed a mechanism to inhibit IFN production by targeting distinct elements of IFN signaling [8]. By attaching to IRF3, viral proteins help coronaviruses in suppressing the host’s innate immune system, by inhibiting the synthesis of IFNß [8–12]. Furthermore, RNA viruses particularly the SARS-CoV and SARS-CoV-2, are prone to rapid mutagenesis that enhanced the virus spread and infection [12].

**Fig. 1:**
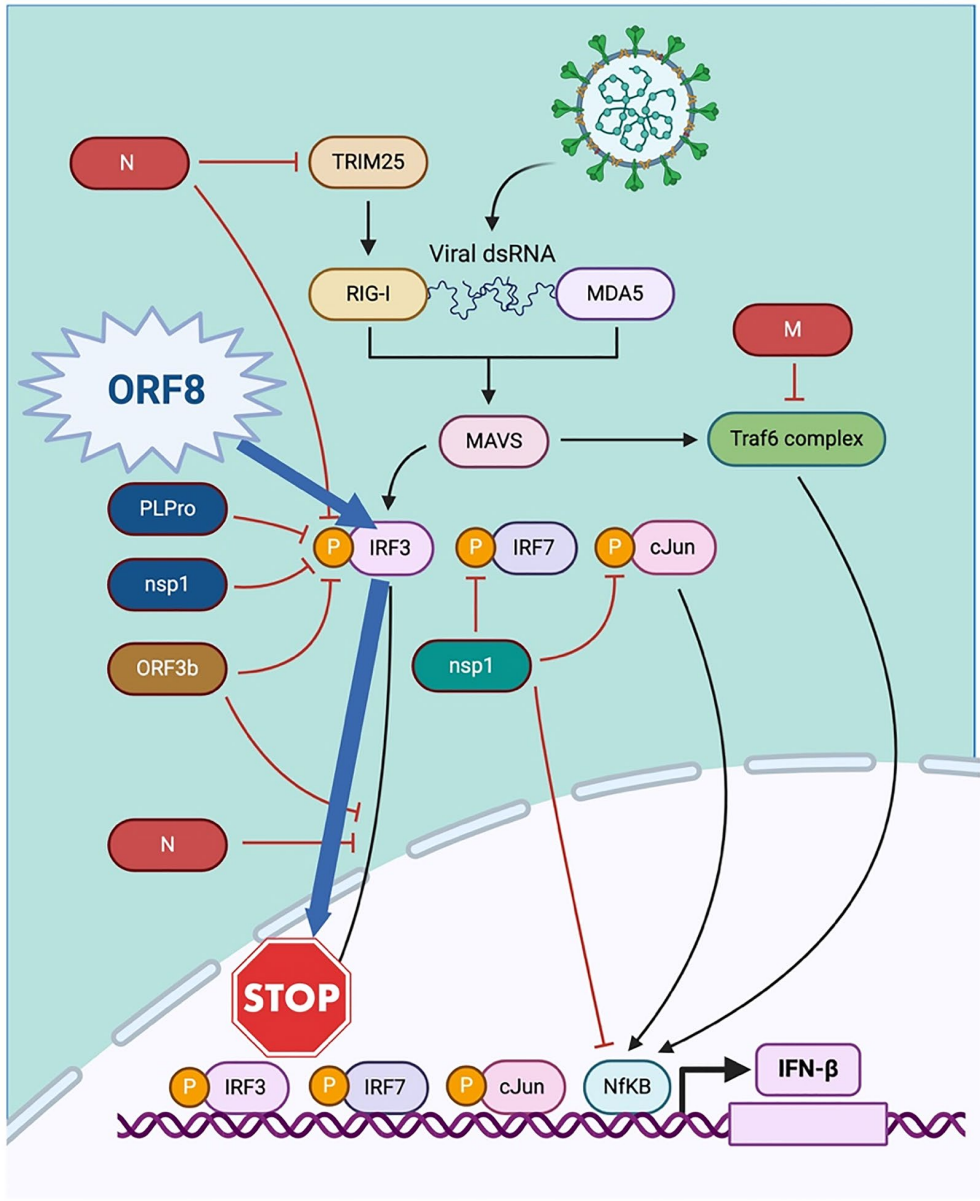
Illustrating the function of ORF8 and IRF3 in immune evasion

ORF8 protein which contains 366 nucleotides and 121 amino acids is the most important SARS-CoV-2 accessory protein, and it is more prone to mutation [13]. ORF8 protein escapes the human immune system by binding to and reducing the nuclear translocation of IRF3 and hence downregulating the MHC-1 (major histocompatibility complex class I) [14] and the type I IFN signaling pathway [15–17]. The pathway is shown in the Fig. 1. Since the appearance of SARS-CoV-2, there have been various mutations detected in ORF8. These mutations, like L84S, V62L, S24L, and W45L, have been identified in various SARS-CoV-2 variants. Changes in ORF8 due to these mutations have influenced their ability to bind with IRF3. For instance, the W45L mutation showed increased affinity for IRF3, suggesting a heightened capacity for immune evasion [18].

Virtual drug screening, molecular dynamics (MD) simulation, and binding free energy calculation collectively revolutionize drug discovery by offering efficient computational methodologies to identify and optimize potential drug candidates[19, 20]. Virtual screening accelerates the initial stages of drug discovery by swiftly evaluating large chemical libraries and prioritizing compounds for experimental testing based on predicted binding affinities [21]. MD simulations provide detailed insights into the dynamic behavior and structural flexibility of biomolecular systems, elucidating key interactions between ligands and their target proteins. Binding free energy calculations complement experimental data by quantitatively estimating the strength of ligand–protein interactions, guiding the rational design of more potent and selective drugs [22, 23]. Carbon nanotubes (CNTs) have a wide range of potential applications, including nanoelectronics, biosensors, biomolecular recognition devices, molecular transporters, cancer therapy, and diagnosis. Many studies have been done on the biological impacts of carbon nanotubes and concluded that the interactions of target proteins and CNTs (nanoparticle–protein corona) play important role in the control of biological activities [24]. Previously the carbon nanotubes were used against the HBx-Bcl-xL interface to halt the hepatitis B viral replication [25]. The same were also used to specifically target the RAN polymerase of the influenza A virus. These investigations revealed that such targeting could effectively impede both the infection by the influenza A virus and the expression of its nucleoprotein and nonstructural protein 1 [26]. The discovery of novel drugs by targeting the interface involved in the pathogenesis makes a significant contribution to the field of therapeutics development [27]. Since ORF8 protein participates in important cellular activities like binding to IRF3 and escaping the human immune system [16]. Therefore, in the current study, the high-affinity carbon nanotubes were used to target the ORF8 interface by using the virtual drug screening, molecular dynamic simulation and binding free energy calculation approaches. Using molecular docking and molecular dynamics simulation we target the interface of ORF8 to curtail the binding of this protein with the IRF3 and reduce the chances of SARS-CoV-2 infection.

## Materials and methods

### Data retrieval and preparation

The crystal structure of the ORF8 protein (PDB ID: 7jtl) was retrieved from the protein databank (https://www.rcsb.org/structure/1p4q) [28]. The GalaxyRefine online server was used for the energy minimization and refinements of the ORF8 protein structure. The GalaxyRefine online server enhances protein structures generated by modeling or prediction methods through a series of refinements including side-chain optimization, loop modeling, energy minimization, and molecular dynamics simulations. By adjusting side-chain conformations and optimizing local geometry, GalaxyRefine aims to improve the overall quality and accuracy of protein models, reducing steric clashes and enhancing packing interactions [29]. To target the ORF8 binding interface we searched the literature and found 62 distinct carbon nanotubes which have the favorable properties and used previously against the biological targets in different diseases [30]. Then the PyRx tool was used for the arrangement and minimization of identified nanotubes [31].

### Screening of carbon nanotubes against ORF8 interface

After preparing all the retrieved nanotubes were tested against the previously identified interface of ORF8 protein used for binding with the IRF3 protein. The residues 62–85 of ORF8 are reported to be responsible for binding with the IRF3 protein. Therefore, these interface residues were selected for binding with the nanotubes. Before commencing virtual screening with EasyDock Vina 2.0, all nanotubes were converted into the.pdbqt format. The ligands were transformed into pdbqt structures, meticulously assigning their atomic charges and atom types using advanced tools like Open Babel. Special attention was paid to non-polar hydrogen atoms, Gasteiger charges, and identifying torsion tree roots necessary for subsequent flexibility analysis of the ligands. On the other hand, preparing the receptor involved generating grid maps with AutoGrid, a crucial step in defining the docking space. This complex process included setting grid dimensions, determining appropriate spacing parameters, and preparing the macromolecule in pdbqt format. Additionally, the receptor was enhanced with hydrogen atoms, charge assignments, and atom type specifications to ensure compatibility with subsequent docking procedures. This software offers an intuitive graphical interface for efficiently screening virtual databases. The screening process utilized the AUTODOCK4 algorithm to evaluate and prioritize potential nanotube candidates. To expedite the initial screening, a lower exhaustiveness setting of 16 was chosen. Following this, the most promising compounds were subjected to a second screening with a higher exhaustiveness value of 64. This phase was undertaken to eliminate false-positive results and reassess the compounds that ranked highest based on their scores [32]. Based on high scores, the best hits were processed for the redocking and rescoring.

### Induced-fit Docking (IFD) of the top hits

In the following phase, the shortlisted compounds with high scores underwent a rigorous screening process known as induced fit docking (IFD), employing an exhaustive 64-step approach. This method utilized AutoDockFR, which utilizes the AutoDock4 scoring function and has been enhanced to improve the success rate of the docking process [33]. During cross-validation docking experiments, it was observed that AutoDockFR exhibited superior accuracy in identifying docking poses and notably outperformed AutoDock Vina in terms of speed without compromising precision.

### Molecular dynamics simulation of the top hits

The molecular dynamics simulation of the selected top hits docking complexes employed the Amber20 package [34]. The simulation incorporated the amber GAFF (general force field) and ff14SB forcefield for the complexes, with drug topologies generated using the antechamber module. Subsequently, each system underwent solvation and neutralization using a TIP3P water box and Na+ counter ions. A two-step energy minimization procedure was utilized, succeeded by a process involving heating and equilibration. The Particle Mesh Ewald (PME) method [35] was employed to calculate long-range electrostatic interactions. Van der Waals contacts and short-range Coulombic interactions were considered within a cut-off range of 1.4 nm. Temperature was maintained at 300 K using a Langevin thermostat, while pressure control utilized a Berendsen barostat. The simulation duration for each complex was 50 ns (50 ns), employing a time step of 2 fs. CPPTRAJ and PTRAJ tools were employed to evaluate dynamics, stability, and other characteristics of the ligand–protein complexes [36, 37].

### Post simulation analysis

The analysis of the tophits-ORF8 complexes involved using CPPTRAJ and PTRAJ software packages to evaluate various structural properties [37]. To assess compactness, dynamic stability, average hydrogen bond formation, and flexibility, specific calculations were employed. Radius of gyration (Rg) was computed to measure structural compactness, employing the formula:

1 $$r_{{{\text{RG}}}}^{2} = \frac{{\sum\nolimits_{i = 1}^{N} {m_{i} \left( {{\mathbf{r}}_{i} - {\mathbf{r}}_{{{\text{CM}}}} } \right)^{2} } }}{{\sum\nolimits_{i = 1}^{N} {m_{i} } }}$$

where ri represents the position of the atom at index i, mi is its mass, rCM is the center of mass, N is the total number of atoms, and r2RG is the squared radius of gyration. To measure structural stability over the simulation, Root Mean Square Deviation (RMSD) was calculated using:

2 $$RMSD = \sqrt {\frac{1}{N}\sum\limits_{i = 1}^{N} {\delta_{i}^{2} } }$$

δ2i refers to the squared disparity between the position of an atom at index i and its position in the reference structure, while N represents the total number of atoms.

For investigating individual residue-level flexibility, Root Mean Square Fluctuation (RMSF) computations were employed. RMSF evaluates residue fluctuations rather than overall complex positional changes. The RMSF was determined via the formula:

3 $$B = \frac{{8\pi^{2} }}{3}{\Delta }r^{2}$$

where B represent the B-factor or thermal factor and Δ**r**^2^ is the mean square deviation. To obtain RMSF while considering the three spatial dimensions, the rearranged equation is used:

4 $$RMSF = \sqrt {\frac{3B}{{8\pi^{2} }}}$$

### The binding free energy calculations

The MMPBSA.PY script was utilized to compute the binding free energy of individual protein–ligand complexes, incorporating 2500 snapshots [38–41]. This approach for estimating free energy is widely adopted in diverse research to measure the total binding energy (TBE) of different ligands across various studies [42–44]. The binding free energy was calculated for receptors (*G*_*receptor, solvated*_), unbound states of ligand (*G*_*ligand, solvated*_) and for each complex (*G*_*complex, solvated*_). Expanding on this, Equation can be expressed as follows:

5 $$\Delta G_{bind} = G_{{\left( {complex, solvated} \right)}} - G_{{\left( {ligand, solvated} \right)}} - G_{{\left( {receptors, solvated} \right)}}$$

To delve deeper into the specific energy contributions, we reformulated Equation as:

6 $$G = E_{Molecular Mechanics} - G_{solvated} - TS$$

To calculate the specific energy term, the formula was restructured as follows:

7 $$\Delta G_{bind} = \Delta E_{Molecular Mechanics} + \Delta G_{solvated} - \Delta TS = \Delta G_{vaccum} + { }\Delta G_{solvated}$$

8 $$\Delta E_{Molecular Mechanics} = \Delta E_{int} + \Delta E_{electrostatic} + \Delta E_{vdW}$$

9 $$\Delta G_{solvated} = \Delta G_{Generalized born} + \Delta G_{surface area}$$

10 $$\Delta G_{surface area} = \gamma .SASA + b$$

The overall binding energy comprises several components. The ΔG_*bind*_ represents the protein–protein, protein–ligand and protein nucleic acid associated free energy. The ΔEMM denoted the overall gas phase energy. Solvation entails the combined effects of polar (ΔGPB/GB) and nonpolar (ΔGSA) components. The polar contribution, ΔGPB/GB, is typically determined through Poisson–Boltzmann (PB) or generalized Born (GB) methods, while ΔGSA represents the nonpolar solvation free energy and is often derived from a linear function of solvent-accessible surface area (SASA).

### Bioavailability, metabolism, and potential toxicity analysis of top hits

For evaluating the Absorption, Distribution, Metabolism, Excretion, Toxicity (ADMET) properties of top hit nanotubes, we employed the admetSAR online tool (http://lmmd.ecust.edu.cn/admetsar2). AdmetSAR is a comprehensive resource providing the latest data on diverse chemicals and their Absorption, Distribution, Metabolism, Excretion, and Toxicity profiles. Its user-friendly interface enables searching by name, CASRN, and similarity. Moreover, admetSAR utilizes the ADMET-Simulator, a cutting-edge chemoinformatics toolbox, to predict approximately 50 ADMET endpoints using highly accurate QSAR models [45].

## Results and discussion

### Structural retrieval and pre-processing

**Fig. 2:**
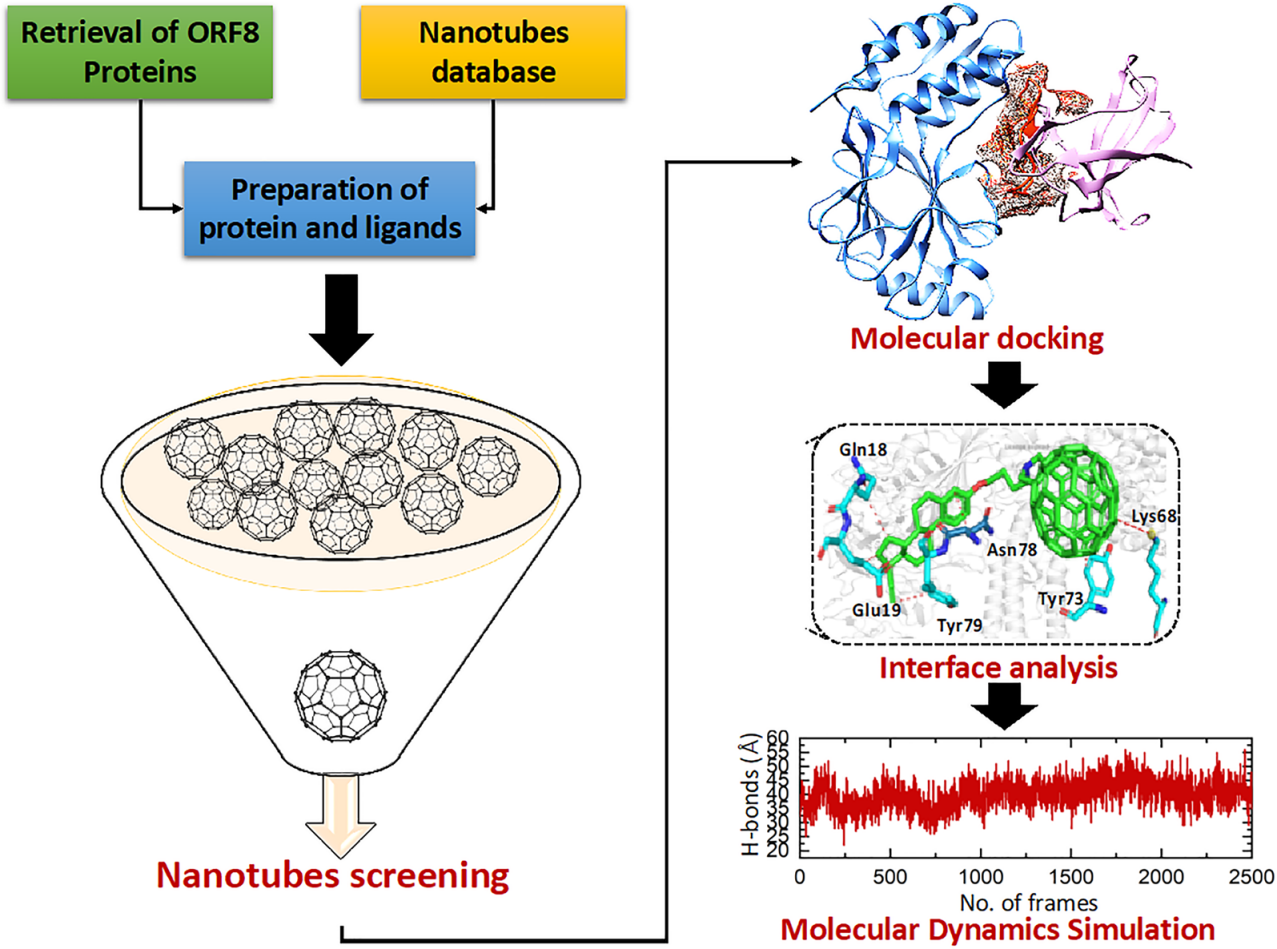
The figure showing the overall work flow of the study such as screening of nanotubes, molecular docking and molecular dynamic simulation

ORF8 protein which contains 366 nucleotides and 121 amino acids is the most important SARS-CoV-2 accessory protein, and it is more prone to mutation [13]. In the human defense system, the activation of the type 1 interferon (IFN) pathway contributes to eliminate viral infection. However, a recent study reported that ORF8 protein escapes the human immune system by binding to and reducing the nuclear translocation of IRF3 and hence downregulating the MHC-1 (major histocompatibility complex class I) [14] and the type I IFN signaling pathway [15–17]. The overall pathway is shown in the Fig. 1. Due to the critical role of ORF8 protein in the human immune system evasion, it could be an important drug target in the therapeutic development of SARC-CoV-2 infection. Therefore, the current study was designed to screen the high-affinity carbon nanotubes against the ORF8–IRF3 interface to block its binding and halt immune evasion by SARC-CoV-2. The 3D structure of ORF8 (PDB ID: 7JX6) was retrieved from the protein databank (https://www.rcsb.org/structure/1p4q) [28]. Then the structure was minimized and prepared before the screening of carbon nanotubes. The overall work flow of this study is shown in the Fig. 2.

### Screening of ORF8–IRF3 binding interface with carbon nanotube

**Fig. 3:**
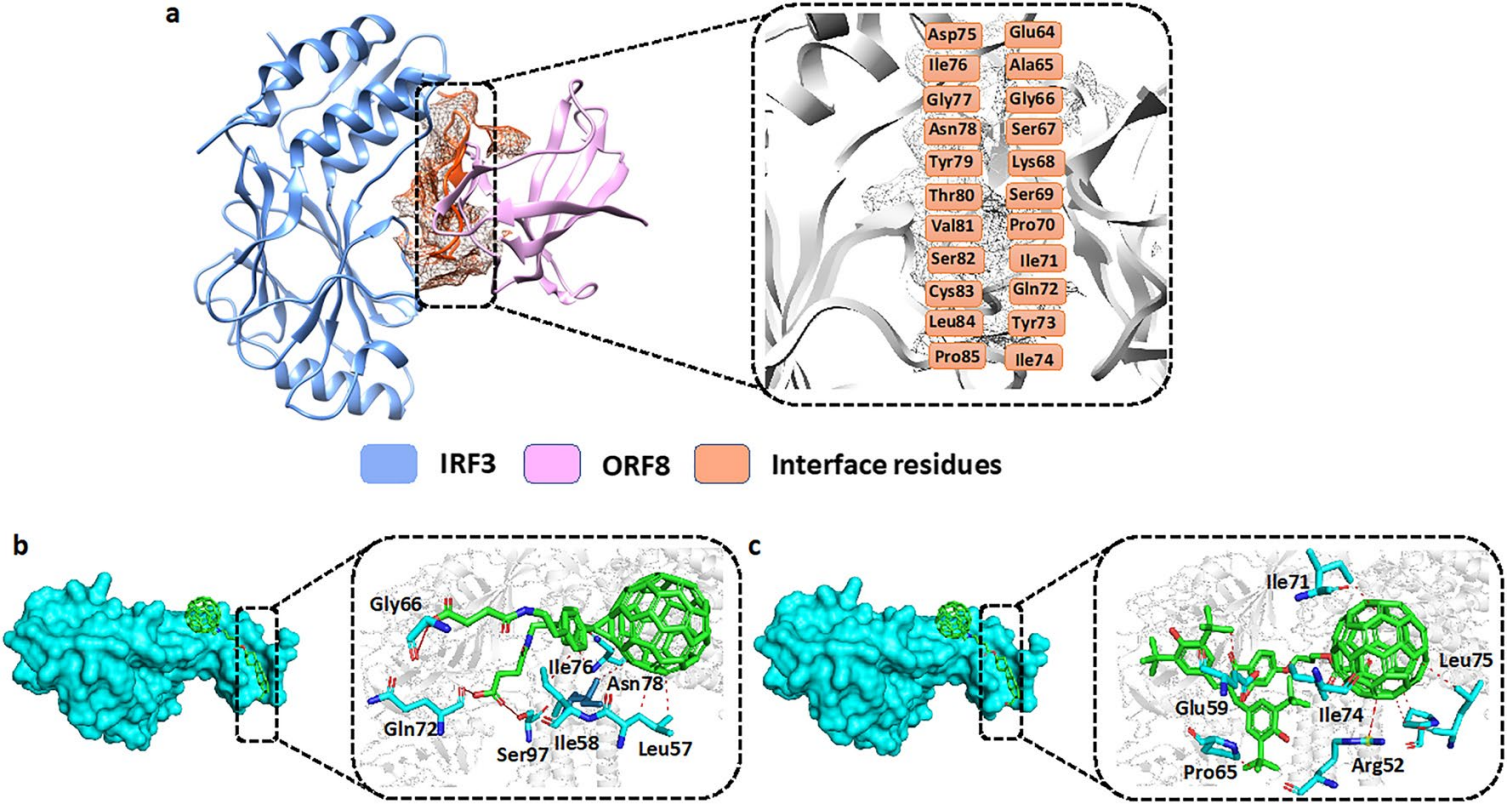
**a** showing interface residues of ORF8 with IRF3. **b** Showing the interactions of best hit1 with ORF8 residues. **c** Showing the interactions of best hit2 with ORF8 residues. The green color represents the best hits while the cyan color represents the interacting residues of ORF8

Carbon nanotubes (CNTs) possess extensive potential applications spanning nanoelectronics, biosensors, biomolecular recognition devices, molecular transporters, and cancer diagnosis and therapy [46]. Numerous investigations have focused on the biological effects of carbon nanotubes, revealing that the interplay between CNTs and target proteins (referred to as nanoparticle–protein corona) significantly influences biological functionalities [47, 48]. A total of 62 carbon nanotubes (fullerenes) were collected from various sources and screened against the ORF8–IRF3 binding interface using the PyRx virtual screening approach. Before docking, the ligand molecules were arranged and the binding interface residues (aa 62–85) of ORF8 were defined for the docking as shown in Fig. 3a. The two-step approach was used for the screening of collected nanotubes with ORF8 proteins. In the first step, the score range was set to − 7.41 to − 4.36 kcal/mol, and screened all the 62 carbon fullerenes. Then in the next step, the induced-fit docking approach with 64 exhaustiveness was carried out to screen the 13 highest scoring compounds to confirm the best final hits. For IDF, to improve the success rate of docking, the ADFR (AutoDcokFR-AutoDock for Flexible Receptor) which utilized the AutoDock4 algorithm was used. The ADFR lowers the internal energy of the receptor and also optimized the confirmation of the receptor side chain. Finally, for shortlisting the best final hits the range of binding energy was set to > − 7.0 kcal/mol. By using the aforementioned parameters, the IFD docking found only 5 compounds having binding free energy > − 7.0 kcal/mol. The docking scores of the first two best compounds were reported to be − 7.94 and − 7.92 kcal/mol, respectively. As shown in Fig. 3b the best hit 1 formed 3 hydrogen bonds with Gly66, Gln72, Ser97, and 6 hydrophobic bonds with the Gly66, Gln72, Ser97, and Leu57, Ile58, Ile76, Asn78 residues. However, the best hit 2 (Fig. 3c) established 1 hydrogen bond with Arg52 and 7 hydrophobic bonds with, Glu59, Pro65, Ile71, Ile74, and Leu75 residues. In conclusion, both the top hit 1 and 2 targeted the specific residues involved in the interaction with the IRF3. For instance, the top hit 1 targeted the Gly66 and Gln72, while Hit 2 targeted Pro65, Ile71, and Ile74 key residues crucial for the ORF8–IRF3 interaction. This targeted binding may impede ORF8's interaction with IRF3 by blocking these critical residues. The interactions of compounds 1 and 2 with the ORF8 residues are shown in Fig. 3.

**Fig. 4:**
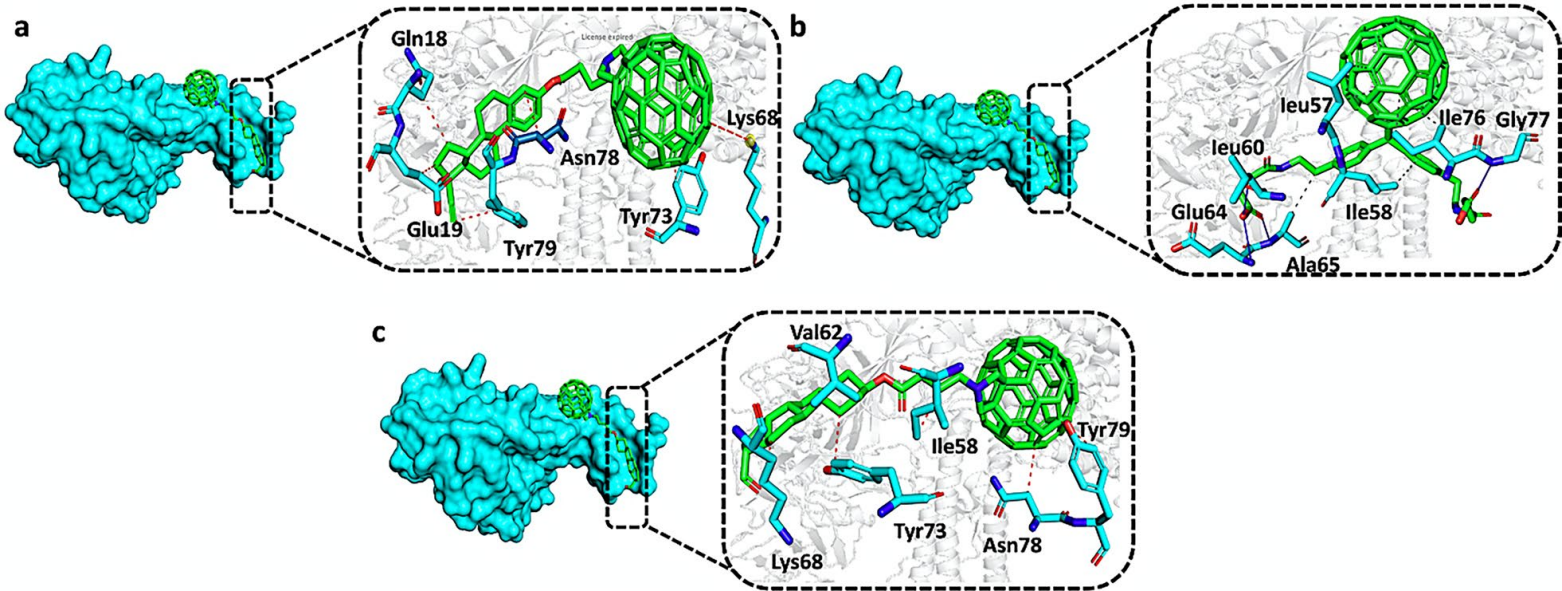
**a** Showing the interactions of best hit3 with ORF8 residues. **b** Showing the interactions of best hit4 with ORF8 residues. **c** Showing the interactions of best hit5 with ORF8 residues. The green color represents the best hits while the cyan color represents the interacting residues of ORF8

Afterward, compound 3 (best hit 3) with a docking score of − 7.28 kcal/mol showed a similar pattern of contacts with the ORF8 binding interface with 1 cation (Lys68) and 5 hydrophobic interactions (Gln18, Glu19, Tyr73, Asn78, Tyr79). Moreover, the docking score of best hit4 was reported to be − 7.19 kcal/mol and established 4 hydrogen (Leu60, Glu64, Ala65, Gly77) and 6 hydrophobic bonds (Leu57, Ile58, Ala65, Ile76) with the ORF8 interface residues. Finally, with a docking score of − 7.09 kcal/mol the best hit5 established 6 hydrophobic interactions with Ile58, Val62, Lys68, Tyr73, Asn78 and Tyr79 amino acid residues. In summary, theses top hits also targeted the specific residues of ORF8 such as Glu64, Ala65, Lys68, and Tyr73 that are involved in the binding with IRF3. Our results revealed high specific binding of the shortlisted nanotubes with the interface residues of ORF8 which may lead to the inhibition of the interaction between the ORF8 and IRF3. The binding network of best hits 3, 4, and 5 with the residues of the ORF8 interface are shown in Fig. 4.

### Evaluation of dynamic stability of ORF8-nanotube

**Fig. 5:**
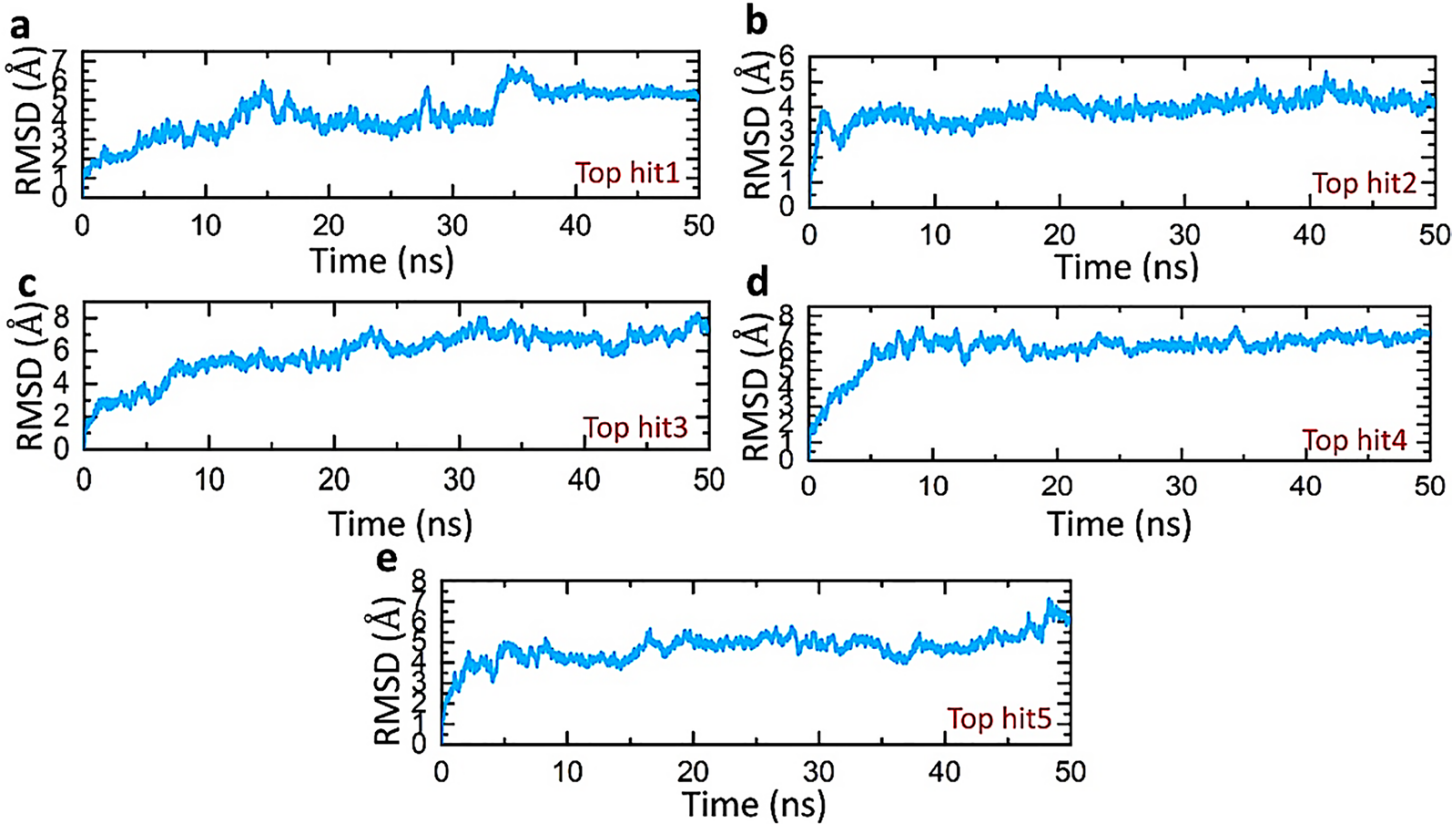
Dynamic stability analysis of top hits-ORF8 complexes (**a**) represents the RMSD value of top hit 1-ORF8 complex (**b**) represents the RMSD value of top hit 2-ORF8 complex (**c**) represents the RMSD value of top hit 3-ORF8 complex (**d**) represents the RMSD value of top hit 4-ORF8 complex (**e**) represents the RMSD value of top hit 5-ORF8 complex

The stability of molecular interactions within a binding cavity is a critical factor in finding the binding efficiency of small ligand molecule. This metric provides details on the dynamic stability of interacting molecules, which can shed light on the binding strength. Understanding a protein’s dynamic stability is crucial in estimating the stability of biological complex in dynamics environment [49, 50]. To test the stability of ORF8-nanotube complexes in a dynamic environment 50 ns simulations were performed. The CPPTRAJ module of AMBER was applied to calculate the stability of complexes by several statistical parameters. For the said purpose, the RMSD (root mean square deviation) was calculated to check the actual deviation of a carbon atom from the original confirmation of docked complexes. The lower RMSD represents the higher complex stability whereas the higher RMSD shows lower stability. The average RMSD for the top hit 1–5 was 4.0 Å, 3.5 Å, 5.2 Å, 6.0 Å, and 4.0 Å respectively. According to the RMSD values, all complexes showed good stability with no major perturbations till 50 ns. The RMSD plot of top hit 1 was uniform until 33 ns with a minor perturbation at 15 ns however, a sudden rise of RMSD was depicted at 34 ns and then gained stability at 40 ns until 50 ns (Fig. 5a). The top hits 2 and 4 gained stability at 5 ns with a mean RMSD of 3.5 Å and 6 Å. Both complexes showed the highest stability with no global or local structural changes noticed until 50 ns (Fig. 5b, d). In the case of the top hit 3 the system equilibrated at 10 ns and then a steady increase was observed in the RMSD valued till 50 ns, however, there is no major fluctuations were seen after equilibration (Fig. 5c). Finally, the top hit 5 complexes gained stability at 5 ns and remained stable until 45 ns with no structural changes which are followed by minor deviation till 50 ns (Fig. 5e). The top hit 2, 4, and 5 showed a greater intermolecular affinity of ORF8 for the respective nanotubes and further validate the result of docking in terms of lowest energy and conformational stability. Figure 4 shows the RMSDs of all complexes.

### Residual fluctuations analysis of top hits-ORF8 complexes

**Fig. 6:**
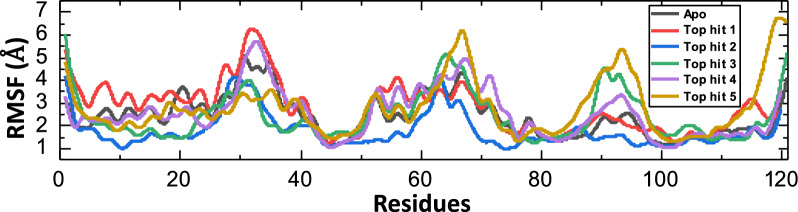
Residues flexibility of all complexes calculated as RMSF

To identify the critical residues important for retaining the interacting ligand and overall stability of complex, understanding the systems flexibility at the residue level is indispensable. Understanding the system's flexibility at the residue level is critical for identifying residues that are critical for retaining the interacting ligand and overall complex stability [51, 52]. To analyze the residues fluctuation, we investigated the RMSF (root-mean-square fluctuation) of the target system. By comparing the Root Mean Square Fluctuation (RMSF) values of a protein with and without a drug, scientists can glean valuable insights into how the drug interacts with the protein and how it affects the protein’s form and function. For instance, when the RMSF values of a protein are elevated in the presence of a drug, it could suggest that the drug triggers structural adjustments and enhances the protein's adaptability [53, 54]. Upon analyzing our findings, we observed an elevation in the Root Mean Square Fluctuation (RMSF) of residues within the target site (80–100) following nanotube binding compared to the apo state. This suggests that the drug's binding induces heightened flexibility or movement in these residues, potentially to accommodate the ligand. The average RMSF value of all complexes is about 2 Å which further confirmed the results of RMSD and affirmed the high stability of all complexes. However, the residues present at N-terminal and C-terminal are showing instability which might be due to the highly flexible nature of protein ends [55]. Figure 6 shows the RMSF values of apo and all complexes.

### Structural compactness analysis of top hits-ORF8 complexes

**Fig. 7:**
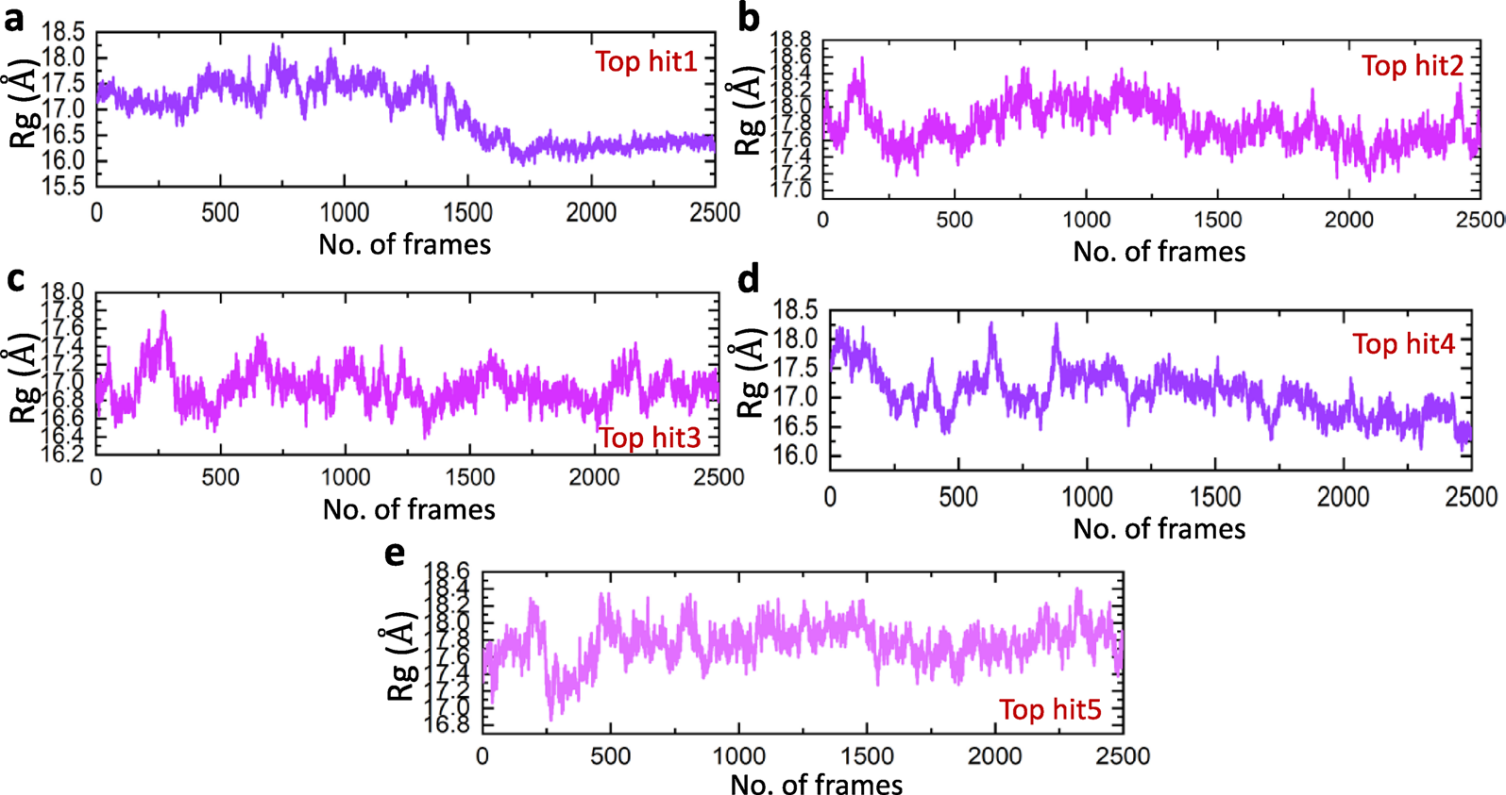
Calculation of Rg for all complexes. **a** showing the Rg value of top hit 1, **b** showing the Rg value of top hit 2, **c** showing the Rg value of top hit 3, **d** showing the Rg value of top hit 4, **e** showing the Rg value of top hit 5

The radius of gyration serves as a crucial metric for assessing the interplay between a protein and a drug molecule. A reduced radius of gyration signifies a more compact arrangement of the protein and drug when they are bonded. This implies that the protein undergoes structural alterations upon binding with the drug, resulting in a tighter, more condensed structure [56]. It offers valuable information regarding the general configuration and density of the protein-drug combination. Therefore, analyzing the radius of gyration in a protein-drug complex can help gauge the effectiveness of drug binding and its subsequent influence on the protein’s conformation [57]. To analyze the structural compactness of protein during the molecular dynamic simulation, we employed the Rg (radius of gyration) analysis. The Rg analysis helped to identify whether the interacting residues in ORF8-nanotube complex are in equilibrium with a lower energy state and whether the residues are tightly bound to each other. Lower the Rg value higher will be the stability whereas a higher Rg indicates the instability of the system. As shown in Fig. 7a–e the average Rg values for the tophit1-5 were found to be 16.5, 17.7, 16.8, 16.6, and 17.6 respectively, however, some fluctuations in Rg values were found during the simulations due to the binding and unbinding of nanotubes with the ORF8 during this time.

### Determining strength of intermolecular interactions

**Fig. 8:**
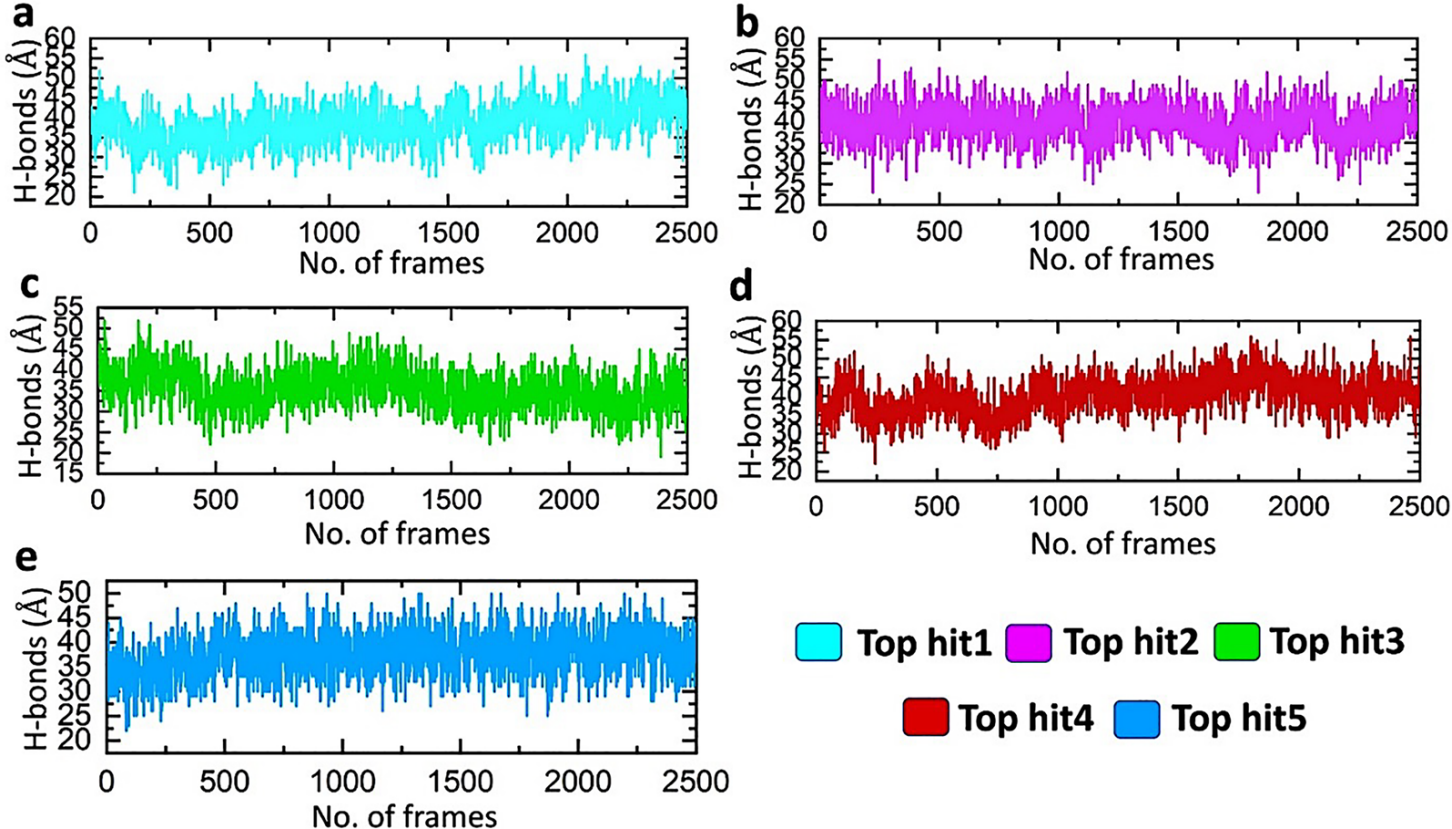
The hydrogen bonding network of all complexes. **a** showing the average hydrogen bonds for top hit 1-ORF8 complex, **b** showing the average hydrogen bonds for top hit 2-ORF8 complex, **c** showing the average hydrogen bonds for top hit 3-ORF8 complex, **d** showing the average hydrogen bonds for top hit 4-ORF8 complex, **e** showing the average hydrogen bonds for top hit 5-ORF8 complex

Hydrogen bonding plays a crucial role in the stability of binding complexes and the effective functioning of biological interactions. Evaluating the hydrogen bonds established in molecular interactions is a valuable method for assessing binding affinity [58]. A comprehensive comprehension of the hydrogen bonding configurations within protein-drug interactions is vital for making precise predictions about the strength of these interactions [59, 60]. The final ORF8-nanotubes complexes were subjected to the hydrogen bonding analysis to calculate the number of hydrogen bonds in the frame during the MD simulations. As shown in Fig. 8 all the ORF8-nanotubes complexes have a strong network of hydrogen bonds which represent the stabile interaction of nanotubes with the target ORF8. In each frame, the average hydrogen bonds in each complex were recorded to be 45, 43, 38, 43, and 40 respectively. The above results of hydrogen bonding counter verify the results of RMSD and RMSF in terms of complex stability. Figure 8 shows the hydrogen bonds of all complexes.

### Binding free energy calculations of top hits-ORF8 complexes

Assessing the binding energy is crucial for re-evaluating the binding conformation and verifying the accuracy of the interacting components. This approach offers superior accuracy and is cost-effective compared to wet lab experiments. Previously, it has been utilized to ascertain the binding energy of small molecules that target receptors and druggable proteins of SARS-CoV-2 [55, 61, 62]. Given the method's broad applicability, we’ve computed the binding free energy for the top five candidates utilizing MD trajectories. The computed Van der Waals energy values for the top-ranking complexes, specifically complexes 1 through 5, demonstrated a distinct stability stemming from intermolecular forces. These recorded values were as follows: − 49.49 ± 1.09 kcal/mol, − 55.58 ± 1.19 kcal/mol, − 55.43 ± 0.69 kcal/mol, − 54.50 ± 0.71 kcal/mol, and − 41.84 ± 0.84 kcal/mol for their respective complexes. These figures emphasize the attractive forces generated by Van der Waals interactions among the molecules, shedding light on the stability and affinity of these complexes. In addition, the electrostatic energy calculations for these same complexes provide insights into their charged interactions. The computed electrostatic energy values are as follows: − 1.93 ± 0.80 kcal/mol, 58.72 ± 2.37 kcal/mol, − 5.52 ± 0.52 kcal/mol, 26.23 ± 2.66 kcal/mol, and 118.97 ± 9.71 kcal/mol, revealing the extent to which electrostatic forces contribute to the overall binding energy. Furthermore, the binding free energies for ESURF, EGB, Delta G Gas, and Delta G Solv have been determined and are presented in Table 1. The total binding free energy for each hit was calculated to be − 43.21 ± 0.90 kcal/mol for hit1, − 41.17 ± 0.99 kcal/mol for hit2, − 48.85 ± 0.62 kcal/mol for hit3, − 43.49 ± 0.77 kcal/mol for hit4, and − 31.18 ± 0.78 kcal/mol for hit5. The total binding energy is mainly contributed by the vdW while in some complexes the electrostatic energies were observed to contribute to the total binding energy. Similar range of values has been previously reported for the drug-protein interactions which further validate our results of binding free energy [20, 27, 63].

**Table 1:** Binding free energy computation for the top hits using MM/GBSA method

Parameters	Top hit 1	Top hit 2	Top hit 3	Top hit 4	Top hit 5
VDWAALS	− 49.49 ± 1.09	− 55.58 ± 1.19	− 55.43 ± 0.69	− 54.50 ± 0.71	− 41.84 ± 0.84
EEL	− 1.93 ± 0.80	58.72 ± 2.37	− 5.52 ± 0.52	26.23 ± 2.66	118.97 ± 9.71
EGB	13.35 ± 0.49	− 39.76 ± 2.01	18.62 ± 0.55	− 10.15 ± 2.46	− 103.63 ± 8.86
ESURF	− 4.74 ± 0.03	− 4.19 ± 0.03	− 4.50 ± 0.03	− 5.05 ± 0.08	− 4.68 ± 0.12
DELTA G gas	− 51.82 ± 0.64	2.79 ± 1.73	− 62.96 ± 0.69	− 28.27 ± 2.85	77.12 ± 9.46
DELTA G solv	8.60 ± 0.52	− 43.96 ± 1.99	14.11 ± 0.53	− 15.21 ± 2.47	− 108.31 ± 8.80
DELTA TOTAL	− 43.21 ± 0.90	− 41.17 ± 0.99	− 48.85 ± 0.62	− 43.49 ± 0.77	− 31.18 ± 0.78

### Analysis of ADMET properties for the selected top hits

ADMET properties analysis is essential for drug design as it enables early identification of compounds with favorable pharmacokinetic and toxicological profiles, reducing the risk of late-stage failures and optimizing drug efficacy. By assessing factors such as absorption, distribution, metabolism, excretion, and toxicity, researchers can enhance drug safety, comply with regulatory standards, and predict clinical outcomes more accurately [64, 65]. Therefore, we proceeded to assess the ADMET properties of our chosen leading compounds utilizing the AdmetSAR online tool. Upon conducting oral bioavailability analysis, it was discerned that top hit 1 and top hit 4 possess the desirable attribute of oral bioavailability, while the remaining candidates lack this property. Notably, the efficacy of drug absorption hinges on water solubility, with heightened solubility indicating enhanced absorption features and heightened bioavailability [66]. Analysis of water solubility indicated that all the top hits displayed remarkably high solubility in water, achieving scores of − 4.173, − 3.889, − 2.964, − 4.173, and − 3.153 for hits 1 through 5, respectively. Notably, all these top hits exhibited exceptional intestinal absorption rates of 100%. While most top hits effectively penetrated the blood–brain barrier, top hit 2 stood out as an exception. It is noteworthy that none of the identified top hits demonstrated carcinogenicity, eye irritation, nephrotoxicity, or skin sensitization. However, both top hit 1 and top hit 4 were associated with hepatotoxicity. Additionally, respiratory toxicity was observed across all top hits except for top hit 2. Despite the importance of the organic cation transporter 2 (OCT2) substrate in renal clearance enhancement, none of the compounds demonstrated activity as OCT2 substrates. Comprehensive ADMET properties of the top hits are summarized in Table 2.

**Table 2:** Bioavailability, metabolism, and potential toxicity analysis of top hits

ADMET properties	Top hit 1	Top hit 2	Top hit 3	Top hit 4	Top hit 5
Oral bioavailability	Yes	No	No	Yes	No
Water solubility	− 4.173	− 3.889	− 2.964	− 4.173	− 3.153
Human Intestinal Absorption	Yes	Yes	Yes	Yes	Yes
Blood–Brain Barrier	Yes	No	Yes	Yes	Yes
Carcinogenicity	No	No	No	No	No
Eye irritation	No	No	No	No	No
Hepatotoxicity	Yes	No	No	Yes	No
Nephrotoxicity	No	No	No	No	No
Respiratory toxicity	Yes	No	Yes	Yes	Yes
skin sensitization	No	No	No	No	No
OCT2 substrate	No	No	No	No	No

## Conclusions

In the stride toward combating the SARS-CoV-2, this study harnessed the power of Nano medicine-based approaches to unravel the pharmacological impact of carbon-based nanotubes. A strategic focus on the ORF8 interface emerged as a beacon of hope, a pivotal move in salvaging the host immune response. Within this molecular theater, five Nano carbons took center stage, demonstrating binding propensity against the essential residues—a promising ensemble of viral adversaries met with precision and efficacy.

The study's narrative seamlessly transitioned into the intricate realm of molecular simulations. The binding free energy, a measure of the molecular forces at play, underscored the robust and favorable interactions between the identified Nano carbons and their viral counterparts.

Yet, this culmination marks not a conclusion but a prelude to the next act. The identified compounds, having displayed prowess in the virtual domain, now beckon the tangible realities of in vitro and in vivo validation. The laboratory and living organisms stand as arenas where these Nano protagonists shall undergo rigorous scrutiny, their efficacy and safety scrutinized in the relentless pursuit of a COVID-19 treatment breakthrough.

## Data Availability

The data are available from the corresponding author on reasonable request.
